# Supervised Learning Classifiers for Electrical Impedance-based Bladder State Detection

**DOI:** 10.1038/s41598-018-23786-5

**Published:** 2018-03-29

**Authors:** Eoghan Dunne, Adam Santorelli, Brian McGinley, Geraldine Leader, Martin O’Halloran, Emily Porter

**Affiliations:** 1 Translational Medical Device Lab (tmdlab.ie), National University of Ireland Galway, Galway, Ireland; 2 Department of Electrical & Electronic Engineering, College of Engineering & Informatics, National University of Ireland Galway, Galway, Ireland; 3 Department of Computer Science & Applied Physics, Galway-Mayo Institute of Technology, Galway, Ireland; 4 School of Psychology, National University of Ireland Galway, Galway, Ireland

## Abstract

Urinary Incontinence affects over 200 million people worldwide, severely impacting the quality of life of individuals. Bladder state detection technology has the potential to improve the lives of people with urinary incontinence by alerting the user before voiding occurs. To this end, the objective of this study is to investigate the feasibility of using supervised machine learning classifiers to determine the bladder state of ‘full’ or ‘not full’ from electrical impedance measurements. Electrical impedance data was obtained from computational models and a realistic experimental pelvic phantom. Multiple datasets with increasing complexity were formed for varying noise levels in simulation. 10-Fold testing was performed on each dataset to classify ‘full’ and ‘not full’ bladder states, including phantom measurement data. Support vector machines and k-Nearest-Neighbours classifiers were compared in terms of accuracy, sensitivity, and specificity. The minimum and maximum accuracies across all datasets were 73.16% and 100%, respectively. Factors that contributed most to misclassification were the noise level and bladder volumes near the threshold of ‘full’ or ‘not full’. This paper represents the first study to use machine learning for bladder state detection with electrical impedance measurements. The results show promise for impedance-based bladder state detection to support those living with urinary incontinence.

## Introduction

Urinary incontinence (UI) is the involuntarily discharge of urine^1^, and it affects over 200 million people worldwide^2^. The condition may develop in an individual due to advancing age^3,4^, urinary tract infections^3^, diabetes^3,5^, or due to a neurological disease such as stroke, spinal cord injury, or multiple sclerosis^3,6^. Urinary Incontinence can be associated with urinary tract infections, urinary reflux and even renal damage and failure^7,8^. Urinary incontinence can also severely impact an individual’s quality of life^9–11^. The condition has four primary types: functional, urge, stress and overflow^1^. Mixed urinary incontinence is a combination of urge and stress urinary incontinence^1^.

Currently, bladder volume monitoring is carried out using a bladder ultrasound (2D and 3D) in a clinical setting. A probe is pressed against the patient’s abdomen and a bladder volume is determined from the resultant bladder image. Clinically employed bladder ultrasounds require staff to periodically check to the bladder volume^12^, increasing the burden on the health system. Attempts have been made to create a wearable bladder ultrasound device^12–14^ that would continuously monitor the bladder volume. However, these devices are bulky^14^ and expensive^15^.

Electrical impedance techniques, such as electrical impedance tomography or electrical impedance plethysmography, offer the potential to continuously monitor the bladder volume of patients non-invasively and proactively. Non-invasive electrical impedance (EI) measurement for bladder volume monitoring (BVM) involves placing electrodes on the skin around the pelvis. Small electrical currents are then injected into the pelvic region through a pair of electrodes and the resulting voltages are measured with the remaining electrodes. The captured voltage measurements are then processed and related to the bladder volume. Feasibility and preliminary studies of BVM using EI on humans have been carried out by Kim *et al*.^16^, Leonhardt *et al*.^17^, Liao and Jaw, 2011^8^, Li, *et al*.^7^ and Zariffa *et al*.^6^. The results from these studies have shown a strong correlation between the processed electrical impedance data and the bladder volume.

One alternate approach to determine the bladder volume or the bladder state from the captured data is to use machine learning. This technique does not necessarily require generation of an image in order to interpret the bladder volume, and it advantageously provides an objective result with minimal computational requirements. Schlebusch, *et al*.^18^ employed a machine learning algorithm as part of a study relating the captured measurements and the reconstructed EIT image data to the bladder volume. The authors used a three hidden-layer, feed-forward neural network (NN) to predict the bladder volumes from simulated voltage frames. While the NN performed well under noisy circumstances, the NN had trouble extrapolating outside or near the limits of the bladder volumes used in the training data. The NN also misclassified bladder volumes under ideal noise conditions.

To address the needs of the population with urinary incontinence, knowledge of the precise bladder volume is rarely needed. Instead, a determination of whether the bladder is ‘full’ or ‘not full’ can be used in order to prevent uncontrolled voiding by providing an alert to the wearer or their caregiver. This solution would have the most potential to support adults with urge, functional and mixed urinary incontinence. The solution would also provide a tool to support those who are not able or struggle to identify the sensation of bladder fullness, even though there is no pathology affecting the bladder itself. For example, these populations could include children with nocturnal enuresis, and children with autism or intellectual disabilities who have difficulty achieving toilet training.

Therefore, in this study, the objective is to determine whether bladder state classification using electrical impedance measurement data is feasible and if so, to determine the robustness of supervised-learning classifiers. The tests of classification robustness that we explored included varying the bladder volume, the amount of noise in the collected voltage frames, the urine conductivity, as well as the boundary (i.e., body shape). We also performed the first machine learning classification for BVM using EI outside of numerical simulations, by employing experimental phantom data.

## Data Generation

In this section, we discuss the generation of simulation data for three main test-cases (TCs), which increase in terms of complexity: TC 1) varying the bladder volume; TC 2) varying the bladder volume, and the urine conductivity; and TC 3) varying the bladder volume, the urine conductivity, and the pelvic boundary. Each TC is tested at four different noise levels. We also discuss the collection of experimental phantom data using EI hardware.

## Simulation Data Generation

**Figure 1:**
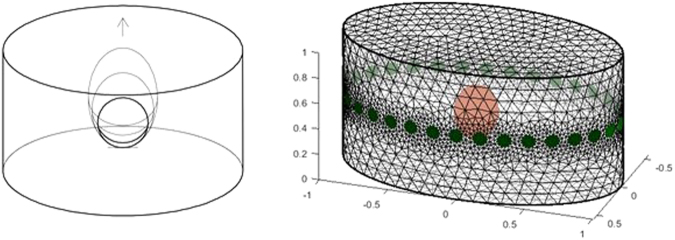
(**a**) Illustration of the bladder ascent into the pelvic region as the bladder volume increases. Note that the bottom position of the bladders is fixed. (**b**) Cylindrical FEM used to aid the forward solve, shown with a bladder volume of 240 ml (green circles indicate electrodes, and red ellipsoid is the bladder).

Simulation data for different bladder volumes was generated using an elliptical cylinder finite element mesh (FEM) with a ring of 32 evenly-spaced electrodes. The mesh was formed using the open-source Electrical Impedance Tomography and Diffuse Optical Tomography Reconstruction Software (EIDORS), version 3.9^19^. For TCs 1 and 2, the horizontal radii of the FEM were proportionally accurate to the axial and coronal radii in a computer tomography (CT) scan from the male candidate in the Human Visible Project^20^ (scale for mm: 1:204.5). The CT scan also contained the urinary bladder. The bladder was modelled as an ellipsoid in the FEM, similar to the method used by Schlebusch, *et al*.^18^. The center of each bladder was placed half way in the anterior region of the FEM. The bottom location of each bladder was fixed as shown in Fig. 1. Fixing the bottom of the bladder mimics the ascension of the bladder into the abdominal region as the bladder fills. To define a fixed bottom location, we determined the empty and the full bladder volumes limits (40 ml and 420 ml, respectively) and the average of 230 ml. The 240 ml bladder volume was the closest bladder volume to the average. Thus, we took the length of the major radius for the 240 ml bladder volume from the centroid of the FEM as the fixed bottom location of the bladder.

Fourteen bladder volumes were chosen between the range of empty and full (40–420 ml, inclusive). As previously mentioned, in many bladder volume monitoring applications, the desired outcome is to alert the device wearer that their bladder is full or near full. Therefore, in this study, we investigated a range of bladder volumes that would be considered ‘full’ and a range of volumes that would be considered ‘not full’. Both ‘not full’ and ‘full’ bladder states comprised of seven bladder volumes. The bladder volumes for the ‘not full’ state started at 40 ml and went in increments of 40 ml to 280 ml. Lukacz, *et al*.^21^ reported an adult bladder functional capacity between 300–400 ml. Thus, the bladder volume threshold, i.e., the threshold between ‘full’ and ‘not full’, was fixed at 300 ml. The bladder volumes for the ‘full’ state were incremented in 20 ml from 300 ml to 420 ml.

Proportionally correct bladder dimensions were required for the study. Dunne, *et al*.^22^ used least square regression lines to predict bladder volumes based on data gathered from 4D ultrasound of 15 healthy male volunteers voiding in the study by Hirahara, *et al*.^23^. The least square regression lines related the axial, the coronal, and the sagittal diameters of the bladder to the bladder volume during voiding. Here, we used the regression lines to determine the radii for the above-mentioned bladder volumes.

A uniform background conductivity of 0.2 S m^−1^ was assigned to the FEM. This background conductivity was based on a weighted average of the pelvic internal organs, muscle and fat from the male human CT scans in the Visible Human project, determined in Dunne, *et al*.^22^. The database of Hasgall, *et al*.^24^ was employed as the source of the conductivities for each tissue. For each bladder volume, the bladder was assigned a conductivity (based on the urine conductivity). The FEM, including the background tissues and the bladder, was then forward solved. Each forward solution resulted in the formulation of an ideal simulation frame (i.e. equivalent to an impedance measurement of the region, taken at a single time instance). The stimulation pattern used in the forward solution had a measurement skip of 4 and injection current of 5 mA_p_ (in accordance with the standard IEC-60601-1^25^). No data was recorded at the injection electrodes, resulting in 928 measurements per frame rather than 1024 measurements. The voltage data generation scripts are available online, please refer to the Data Availability section.

In more complex TCs where the simulated conductivity is varied (TCs 2 and 3), the urine conductivity ranges from 0.5–3.25 S m^−1^, in increments of 0.25 S m^−1^. The conductivity of the thin bladder wall was discounted, and the conductivity of the bladder was considered the same as the conductivity of urine. The urine conductivity range aligns with the range of urine conductivities reported in the current literature^24,26–28^.

In TC 3, along with varying the bladder volume and bladder conductivities, six different boundaries were used for the FEM that were based on male and female CT scan proportions from the Visible Human Project^20^. The horizontal anterior-posterior diameter for both the male and female proportions were varied by ±10% resulting in the 4 additional boundaries.

Four Random Gaussian White Noise (RGWN) levels were applied to each ideal simulation frame for each TC in order to model data recorded from a real-world electrical impedance measurement device. Current literature defines the signal-to-noise ratio (SNR) in dB for modern electrical impedance capture devices as between 30–120 dB^29,30^. For this work, the noise levels employed were {20, 40, 60, 80} dB. An SNR value outside of the expected measurement range (i.e., 20 dB which is less than the worst-case expected measurement SNR of 30 dB) was used to determine how the classifiers performed in severe and unexpected circumstances. We define the RGWN for a single potential, ${u}_{i}$, in the frame of potentials, $u$, as:

1 $${u}_{i,noise}={u}_{i}+{\arg }\,{\max }(u)\ast {\eta }_{i}$$

2 $${\eta }_{i}=\frac{1}{\sigma \sqrt{2\pi }}\exp (\frac{-{({u}_{i}-\mu )}^{2}}{2{\sigma }^{2}})$$

where ${u}_{i,{noise}}$ is the resultant noisy potential value, ${\eta }_{i}$ is the RGWN definition for an individual potential, with the mean $\mu$ and the standard deviation $\sigma$. The mean and standard deviation values are formed as uniformly distributed random values of the SNR.

The number of noisy frames for each ideal simulation frame varied between the three main TCs. The reduction of the number of noisy frames between TCs was due to the size of the dataset increasing with the complexity of the TCs. This lead to longer computational times to train the classifier as well as increased memory storage requirements. The number of noisy frames for each simulation frame in TCs 1, 2, and 3 were 99, 39, and 9 noisy frames respectively. In a real-life measurement, one measurement frame only takes 66.67 ms to collect (at 15 Hz frame collection rate), so in a bladder state detection application it would be possible to have many frames available for classification training/testing. At the same time, having fewer frames makes the classification harder. Thus, if the classifiers are able to perform with fewer frames in the training set, then we would expect the classifier to be stronger when more frames are available. The TCs factors are summarised in Table 1.

**Table 1:** Factors in each of the TCs. For each TC, separate datasets were formed for the SNRs of {20, 40, 60, 80} dB.

Factor	TC 1	TC 2	TC 3
Bladder Volume (ml) [not full state, full state]	[40:40:280, 300:20:420]	[40:40:280, 300:20:420]	[40:40:280, 300:20:420]
Urine Conductivities (S m^−1^)	1.75	[0.5:0.25:3.25]	[0.5:0.25:3.25]
FEM	Male FEM	Male FEM	Male FEM Male FEM ± 10% Female FEM Female FEM ± 10%

The classifier is based on a hard threshold between full and not full. However, to cater for patient specific cases, this threshold may be altered. With functional urinary incontinence where impairments limit the patient reaching the toilet, the not full/full threshold may be reduced so that the patient can proactively go to the toilet, or increased to cater for larger maximum bladder capacities. To verify that the results of the classifier are independent of the volume threshold chosen, we additionally performed a TC with the full/not full separation at 360 ml. All bladder volumes used for not full and full in previous TCs were increased by 60 ml and the new noisy frames were generated. We repeated the factors used in TC 3 with this new separation for the SNR of 20 dB.

Lastly, we generated simulation data for a more realistic scenario (TC 4), using the trained classifiers from TC 3. The realistic scenario dataset consisted of data of withheld SNRs, bladder volumes, urine conductivities, and FEM dimensions. In other words, none of the precise values for SNR, bladder volume, urine conductivity, and FEM dimensions used in the test scenario were seen in the training data. Table 2 shows the withheld factors that are now tested in TC 4. Extra bladder volume observations around the bladder state threshold were also included (295 ml, 299 ml, 301 ml, and 305 ml). The total size of the real scenario test set was 100,584.

**Table 2:** Withheld factors for the realistic scenario dataset (TC 4).

Factor	Withheld Values
SNR (dB)	{30, 50, 70} dB
Bladder Volume (ml)	{20:40:290, 295, 299, 301, 305, 310:20:430}
Urine Conductivities (S m^−1^)	0.625:0.25:3.125
FEM	±5%, ±15% of the original male and female proportions

## Phantom Data Collection

Phantoms have been used previously for electrical impedance measurements in order to bridge the gap between simulation and human data in areas such as breast health classification^31^. Anatomically and conductively accurate phantoms can approximate the human specific region of interest and allow investigation of factors that would be otherwise difficult to isolate. Phantoms also enable refinement and optimisation of clinical factors prior to use of the device in clinical trials.

In our previous work^22^, an anatomically and conductively accurate phantom of the male human pelvis was developed and fabricated for BVM using EI. This phantom used six bladders that were designed to correlate with actual human bladder volumes, allowing for accurate bladder volume estimation. Each component of the phantom corresponds to a tissue or a set of tissues, with electrical properties match to their respective conductivities. The phantom shape was based on computer tomography scans of a male pelvis. A weighted average was used to determine the internal background conductivity and was based on computer tomography scans of the pelvis from the Visible Human Project^20^. Urine has been shown to vary between 0.59–3.22 S m^−1^ and thus, has the highest conductivity in the human body. The weighted average of the background caters for the loss of current through the body (excluding the bladder). Due to the large relative difference between urine and the next high conductivity substance (blood^24^ with a conductivity of 0.7 S m^−1^), this model can suitably provide results that can be extrapolated to actual human conditions.

**Figure 2:**
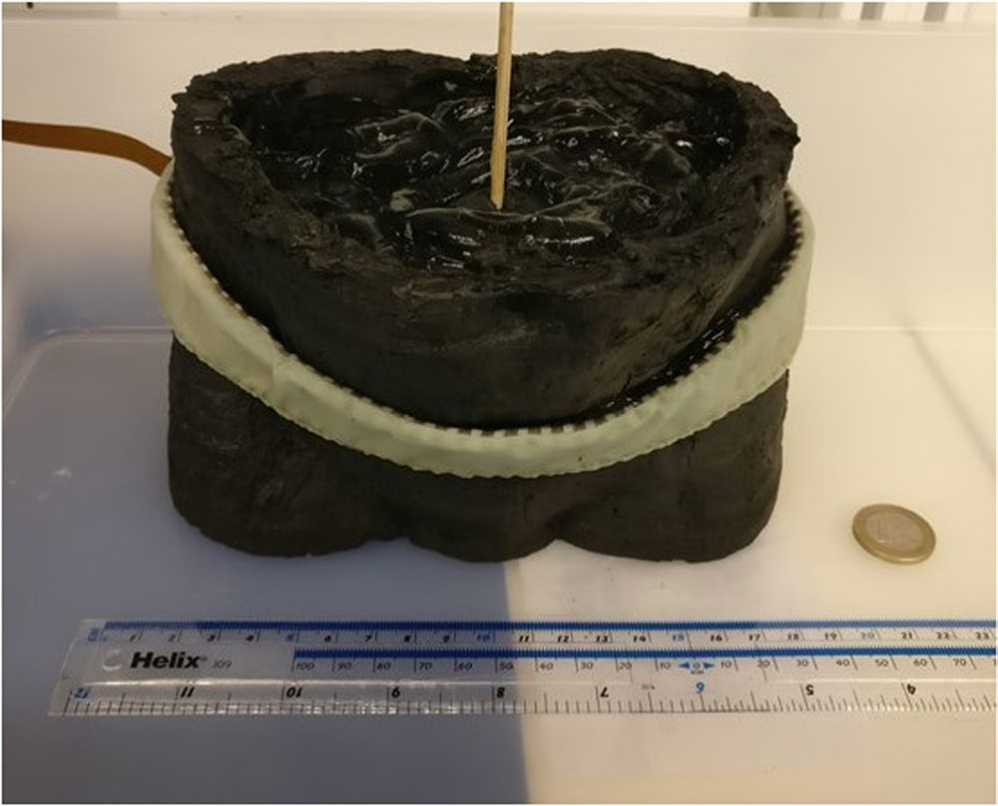
Phantom experimental setup. The bladder is submerged in ultrasound gel within the anterior region of the phantom. Further details on the experimental setup are available in Dunne *et al*.^22^.

Experimental data was recorded using a Swisstom Pioneer Set (Swisstom AG, Landquart, Switzerland) with a 44 cm, 32 electrode belt. In total, 30 seconds of data was captured from the phantom for each bladder volume (40, 60, 100, 160, 240, 400 ml) at 50 kHz. This data, when reconstructed, showed a strong positive trend between bladder volume and the average conductivity index. The phantom experimental setup is pictured in Fig. 2 (and is further described in our prior study^22^). We utilized this experimental data as the source for our classification dataset. The frames recorded for each bladder volume were each considered as an observation. Measurements from the injection electrodes were removed, resulting in 928 measurements per frame.

## Classification Methods

In this section, we discuss the preparation of both the simulation and phantom data for classification, the classifiers employed, their design, and the classification process.

### Data Preparation for Classification

#### Simulation Data Preparation

In simulation, a dataset was compiled for each SNR in each of the three test-cases (TCs), based on the generated simulation data in the Data Generation section. For each measurement (e.g. a specific urine conductivity and bladder volume), the set of frames (i.e., observations) consisted of the ideal simulation frame and the corresponding noisy frames. Table 3 outlines the dataset size for each TC and the number of factors for each dataset.

**Table 3:** Number of factors for the dataset of each test-case (TC).

	TC 1	TC 2	TC 3
SNR levels	4	4	4
Number of Ideal Frames per Scenario	1	1	1
Number of Noisy Frames per Scenario	99	39	9
Bladder Volumes	14	14	14
Conductivities	1	13	13
Boundary sizes	1	1	6
Total Number of Observations	1,400	7,280	10,920

On selection of a particular SNR in a TC, the dataset was divided into ‘full’ and ‘not full’. The observations in ‘full’ and ‘not full’ sets were randomized. Ten training and test sets were then formed for 10-fold testing (see paper by Li, *et al*.^32^ for further detail on 10-fold testing). The training set consisted of 90% of the observations in each of the ‘full’ set and the ‘not full’ set, and the test set consisted of 10% of the remaining observations in each of the ‘full’ and ‘not full’ sets. The observations in a test set were never repeated in another test set.

#### Phantom Data Preparation

All bladder volume observations less than 300 ml were labelled ‘not full’, and bladder volume observations greater than or equal to 300 ml were labelled ‘full’. The total dataset of 900 observations consisted of 450 frames of the ‘full’ volume of 400 ml and 90 frames from the other five ‘not full’ volumes. Similar to the simulation data preparation described previously, the dataset was divided into ‘full’ and ‘not full’. The observations in ‘full’ and ‘not full’ sets were randomized. As with the simulation data, ten training and test sets were then formed for 10-fold testing. The training set consisted of 90% of the observations from each of the ‘full’ set and the ‘not full’ set, and the test set consisted of 10% of the observations from each of the ‘full’ set and the ‘not full’ set. The observations in a test set were never repeated in another test set.

### Design of the Classifiers

This paper proposes a binary supervised learning problem for electrical impedance bladder monitoring. Support vector machines (SVMs) have been shown to be highly effective for binary classification^33^. Support vector machines attempt to separate a set of inputs into two classes using a hyperplane. The simplest form is linear SVM, where a line separates the inputs into two classes. The line is optimized for the best separation of the inputs. Where the inputs are not suitable for linear separation, a specific kernel can be employed to transform not separable inputs in a low-dimensional space into separable data in a higher-dimensional space^33^.

In this paper, we compare SVM classifiers to a *k*-Nearest-Neighbours (KNN) classifier. The KNN classifiers are a part of instance-based learning and are easy to implement. The KNN algorithm stores the training data and on classification, compares the new observation to *k* similar stored observations in order to determine the label of the new observation. To compute the similarity of the observations, a distance metric is employed.

Different types of SVMs and KNNs were tested on the training data for TC 1 using 10-fold validation. Linear and Cubic SVMs, and Cosine KNN performed the best, and as such are the ones focused on in this study. Cubic SVM uses a third-order polynomial as the kernel. This kernel has a hyperplane in a higher dimensional space that separates the data. Cosine KNN employs the cosine distance metric to determine the similarity of a new observation to stored training inputs.

Each classifier was given the 928 voltages in a captured frame as an observation, and, in training, the label (‘full’ or ‘not full’). Each predictor in the observations was standardized. The number of neighbours for the Cosine KNN classifier, *k*, was set at 10 and no distance-weighting was performed.

### Classification Process

**Figure 3:**
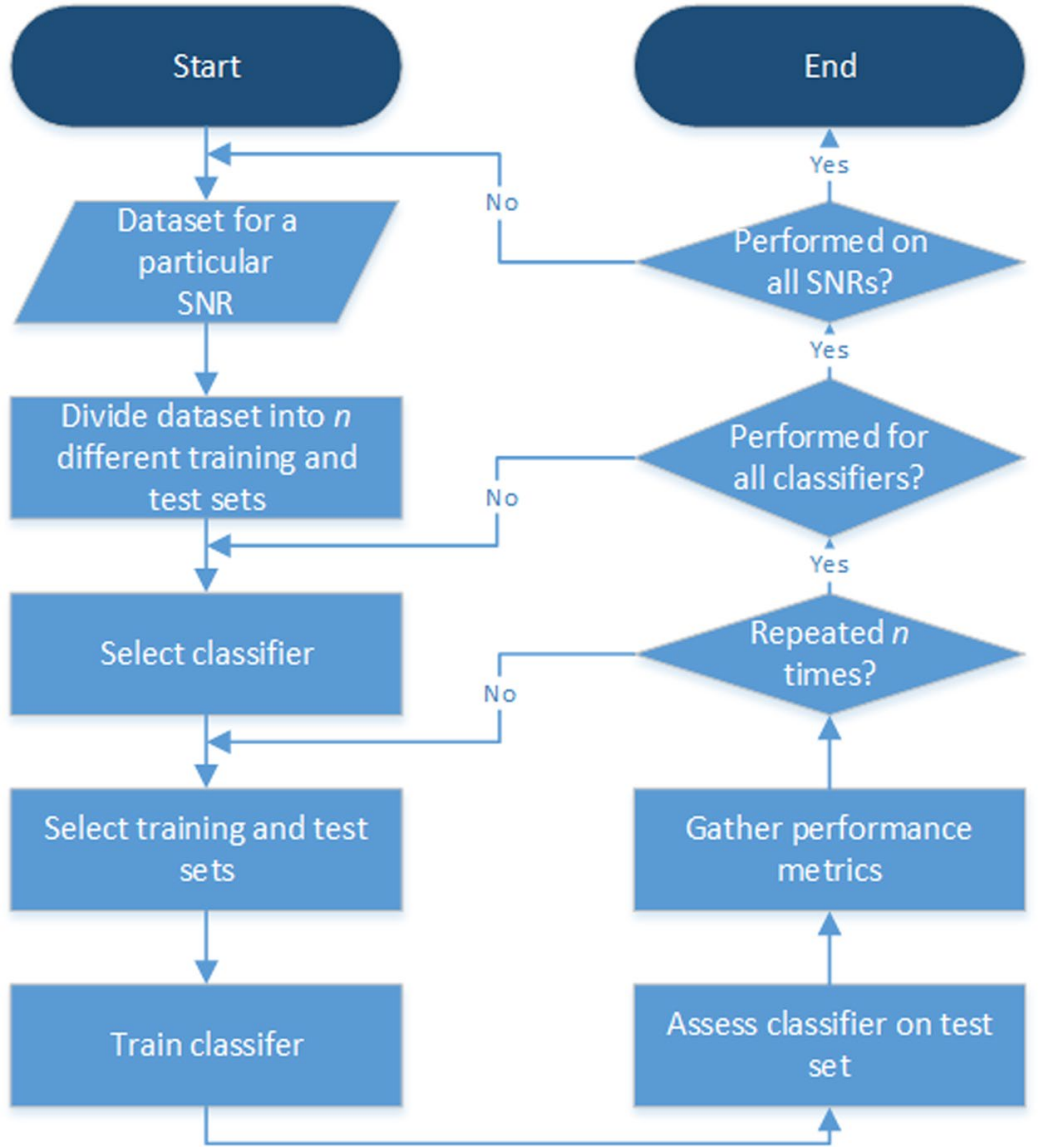
Overview of the classification process. For each dataset with a unique SNR, *n* training and *n* test sets were formed. A classifier for each classifier type was trained on a particular training set and subsequently tested on the corresponding test set. In this paper, *n* is set to 10.

For each SNR and TC, the dataset was divided into 10 training and test sets. The three classifier types were then trained and tested on the same dataset and assessed each time. The assessment of the test set involved the following performance metrics: the accuracy, the sensitivity, and the specificity. The sensitivity is the correct prediction rate of the bladder being full and the specificity is the correct prediction rate of the bladder being not full. The accuracy is defined as the mean of the sensitivity and specificity. The final reported results were computed in terms of the mean and standard deviation of each performance metric, across all test combinations of a given dataset, SNR, and classifier type. Figure 3 illustrates the classification process.

### Data Availability

The voltage data generation scripts are provided online at https://github.com/EoghanDunne/EIBladderStateClassification_1. This paper contains all the information required to generate the datasets and the parameters for the classifiers.

## Results and Discussion

In this section, we report and discuss the classification results from both simulation and phantom experiments using SVM and KNN-based classifiers.

### Simulation Results and Discussion

#### Test-case 1–3

**Figure 4:**
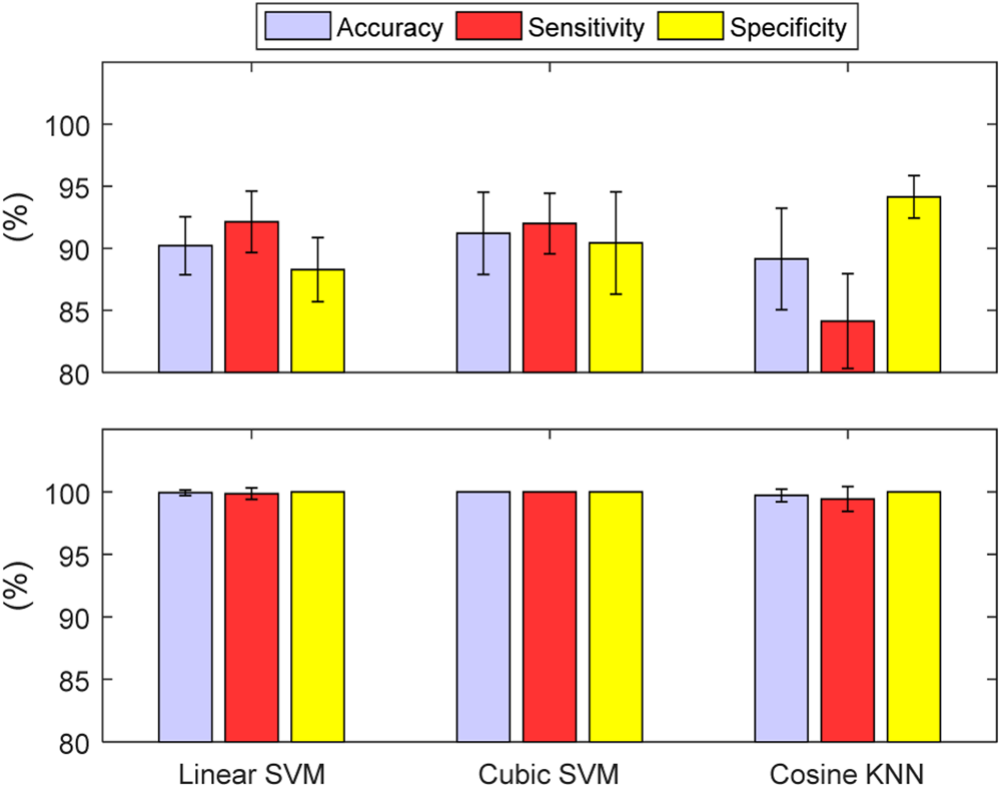
The performance metrics for TC 1 at SNRs of 20 dB *(top)* and 40 dB *(bottom)*. The performance metrics are reported in terms of the mean and standard deviation of the 10 classifiers formed for each of the classifier types: Linear SVM, Cubic SVM and Cosine KNN. As the SNR decreases, so does the performance of the classifiers.

**Figure 5:**
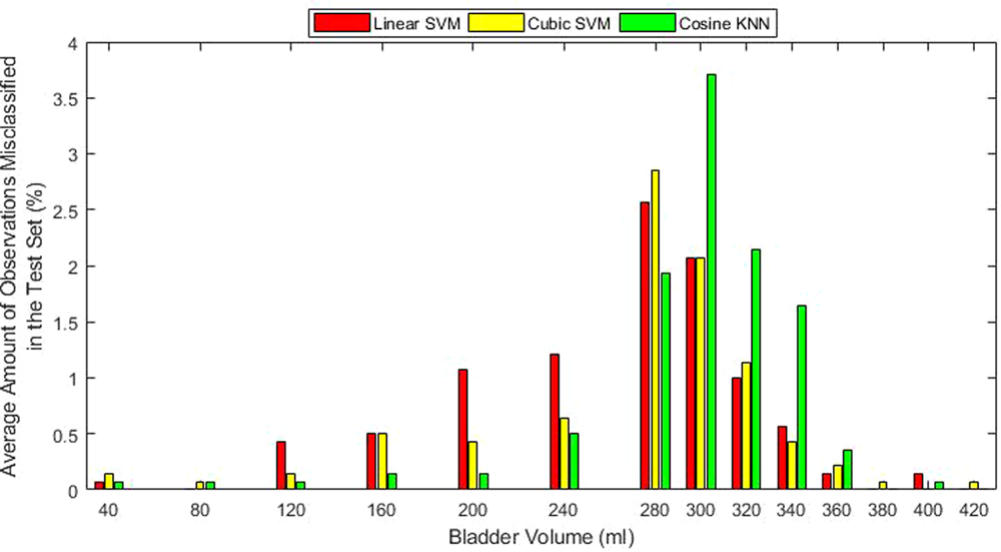
Analysis for TC 1 of the misclassified observations per bladder volume, across each classifier type on the 20 dB test set. Each classifier type had 10 classifiers whose misclassification frequencies were averaged and converted into percentage of the test data size. Most misclassification occurred around the bladder volume threshold of 300 ml.

Test-case 1 involved the simplest scenario of varying the bladder volume for a particular SNR (with all other variables fixed). Four SNRs were tested: {20, 40, 60, 80} dB. At 60 dB and 80 dB, each classifier accurately predicted all the bladder states (100% sensitivity and specificity). As expected, a degradation in performance was observed as the SNR decreased. This degradation is illustrated by the performance metrics in Fig. 4. The degradation of accuracy is slight for 40 dB but quite noticeable in the 20 dB scenario. The errors in the 20 dB scenario for Linear SVM, Cubic SVM and Cosine SVM are 9.71%, 8.79%, and 10.57%, respectively, and are 0.07%, 0%, and 0.29%, respectively, for the 40 dB scenario. Both SVM classifiers have a higher sensitivity than specificity at 20 dB. In contrast, the Cosine KNN classifier has a stronger specificity, but also has lower accuracy compared to the SVM classifiers. We further analysed the misclassified bladder volumes on the 20 dB test set, Fig. 5, as this is where the highest number of misclassifications occurred. The analysis showed that most misclassified bladder volumes were around the threshold (300 ml) of ‘full’ or ‘not full’, as would be expected. From this graph, we can see that cosine KNN suffers from poorer specificity due primarily to bladder volumes of 300 ml, 320 ml, and 340 ml, whereas the two SVM classifiers suffer from poorer sensitivity with the 200 ml, 240 ml, and 280 ml volumes. Misclassification around the threshold may not be serious, especially if a conservative estimate of a full bladder for the threshold is chosen. On the other hand, alerting when the bladder is empty or after voiding has occurred would be problematic.

**Figure 6:**
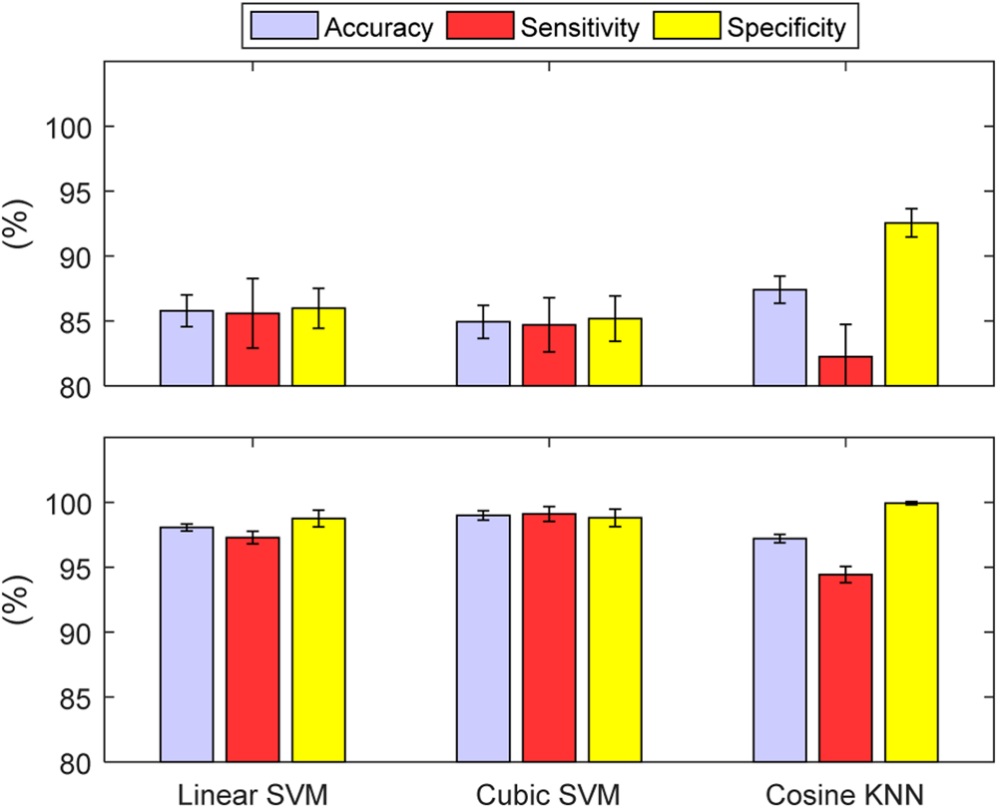
The performance metrics for TC 2 at SNRs of 20 dB *(top)* and 40 dB *(bottom)*. The performance metrics are in terms of the mean and standard deviation of accuracy, sensitivity, and specificity. The performance of each classifiers is reduced by the addition of the new factor, variable urine conductivity. Cosine KNN outperformed the SVM classifiers at 20 dB. Figure 7 provides additional information on the misclassification rates relative to changes in bladder conductivities.

**Figure 7:**
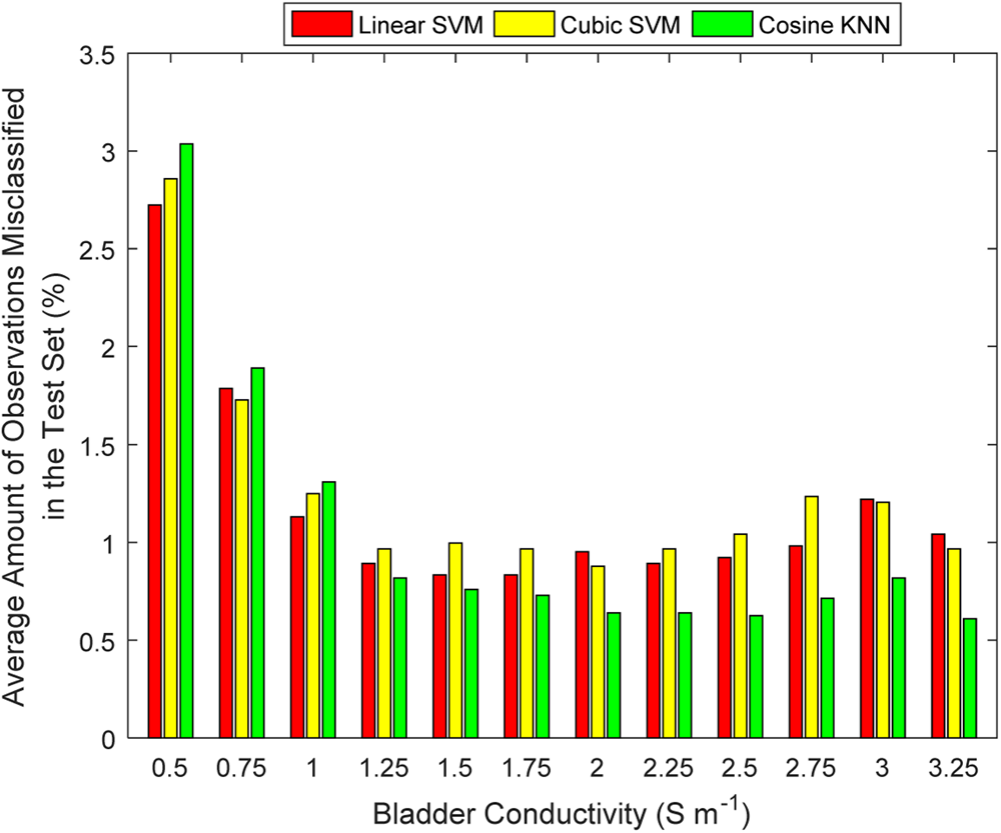
Analysis for TC 2 of the misclassified observations for each urine conductivity, across each classifier type on the 20 dB test set. Each classifier type had 10 classifiers whose misclassification frequencies were averaged and converted into a percentage of the test data size. Most misclassification occurred at lower bladder conductivities, as would be expected.

Test-case 2 built on TC 1 by varying the urine conductivity for each bladder volume. Similar to TC 1, each classifier accurately predicted all of the bladder states in the 60 dB and 80 dB test sets. For both 20 dB and 40 dB test sets, there was a decrease in classifier performance compared to TC 1, with the addition of urine conductivity variance (Fig. 6). Linear SVM produced a stronger specificity than sensitivity on the 20 dB test set, in contrast to TC 1. Also, Cosine KNN had a higher accuracy than the SVM classifiers on the 20 dB test set. Investigation of the misclassified bladder conductivities at 20 dB showed that there was very little difference in the misclassification of bladder conductivities, except at below 1 S m^−1^ (Fig. 7). At lower bladder conductivities, the voltage recordings are higher since the current is less attenuated in the body. Analysis of the mean contrast in voltages between the bladder volumes determined the contrast between the bladder volume measurements is less at lower bladder conductivities. Adding noise to the weak contrasted data, in turn, seemed to lead to more misclassification. However, overall, the misclassification rate was still low (<3.1%), even for the low urine conductivity values. Linear SVM performed the best at lower bladder conductivities while Cosine KNN performed the best at higher bladder conductivities. The misclassification magnitudes and behaviour of the bladder volume were similar to that in TC 1. However, the observations for the 280 ml bladder volume were the most misclassified and Cubic SVM misclassified the most bladder volumes below 280 ml. Linear SVM misclassified the most bladder volumes at 280 ml.

**Figure 8:**
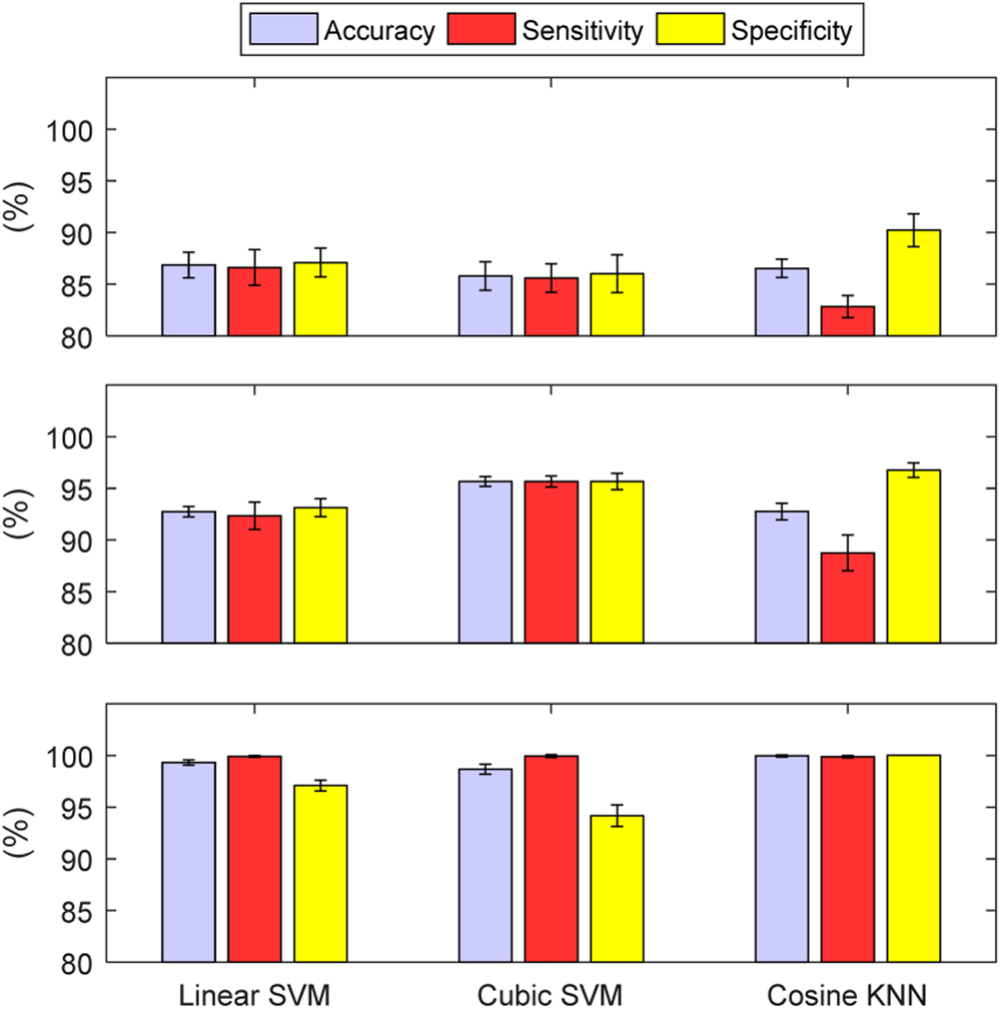
The performance metrics for TC 3 at SNRs of 20 dB *(top)*, 40 dB *(middle)* and 60 dB *(bottom)*. The performance metrics include the mean and standard deviation of accuracy, sensitivity, and specificity. There is only a minor difference in performance for the classifiers at 20 dB in TC 3 compared to TC 2. However, the performance degradation of the classifiers is quite noticeable at 40 dB and 60 dB.

Test-case 3 increased the complexity of the simulation further by changing the FEM boundary, mimicking the change from subject to subject (i.e. body size/shape) on which the measurements are recorded. In TC 3, only the classifiers using the 80 dB test set were able to accurately predict each bladder state correctly. The performance measures are illustrated in Fig. 8. The addition of the boundary change had only a minor effect on the 20 dB classifiers but brought the accuracy of the 40 dB Linear SVM and Cosine KNN classifiers below 94%. The classifiers performed very similar to each other for both the male and female modelled boundaries. When the boundary size was increased, the classifiers misclassified the most observations. As the boundary was brought nearer to the bladder than the original proportions (i.e. the boundary size was decreased), the misclassification of observations was reduced. The classifiers performed similarly with only 0.30% difference in misclassification rates across all observations (1,092 observations in the test set). For the most amount of FEM variations, Linear SVM and Cosine KNN performed the best. The maximum contribution of a FEM variation to the misclassification of test set observations, measured across the classifiers, was 2.73% of the test set size. The results of TC 3 reinforced the trends and contributions of the factors from TCs 1 and 2 on the misclassification of the test set observations. The observations for 280 ml bladder volumes were the most misclassified across the bladder volumes and no substantial change was determined with the urine conductivity histogram.

With each TC, we increased the complexity of the scenario. We found the SNR and bladder volume around the bladder state threshold had the largest effect on misclassification. Changing the FEM had the smallest overall influence on misclassification rates compared to SNR and the near threshold bladder volumes. The SVMs performed consistently well across the TCs, particularly in noisy scenarios. The lowest accuracy observed from any of the three classifiers during the TCs and across the noise levels was 84.94 ± 1.28%, highlighting the potential of bladder state classification using supervised learning.

#### Altering the Threshold

To verify that the classification results are independent of the bladder separation volume, we tested the factors in TC 3 with new classifiers using a threshold of 360 ml for the SNR of 20 dB. The bladder volumes previously used in TCs 1–3 were increased by 60 ml. The accuracies for Linear SVM, Cubic SVM and Cosine KNN were 87.06 ± 0.45%, 84.72 ± 1.12%, 87.67 ± 0.76%, respectively, which are similar to accuracies observed for each classifier in Fig. 8, within 1%. This result is particularly important as the level of fullness is patient and condition specific. Hirahara *et al*. (2005)^23^ showed how urine volumes in the bladders of a sample of healthy volunteers before urination varied from 100 ml to around 380 ml. Individuals suffering from urinary retention can have also distorted bladders containing more than 450 ml in volume^34^. Thus, we can modify the threshold between full and not full based on patient specific requirements without affecting the accuracy.

#### Test-case 4: Realistic Application Scenario

The final simulation test was a more realistic scenario, where the classifiers were tested on one test set that included SNRs, bladder volumes, bladder conductivities, and FEM dimensions withheld from the classifiers previously. These factors are outlined in the Data Generation section. We employed the trained classifiers formed in TC 3, that were originally trained and tested on the 40 dB dataset. The resulting overall accuracies of the Linear SVM, Cubic SVM, and Cosine KNN were 81.66 ± 0.12%, 73.16 ± 0.74%, and 79.98 ± 0.35%, respectively.

**Figure 9:**
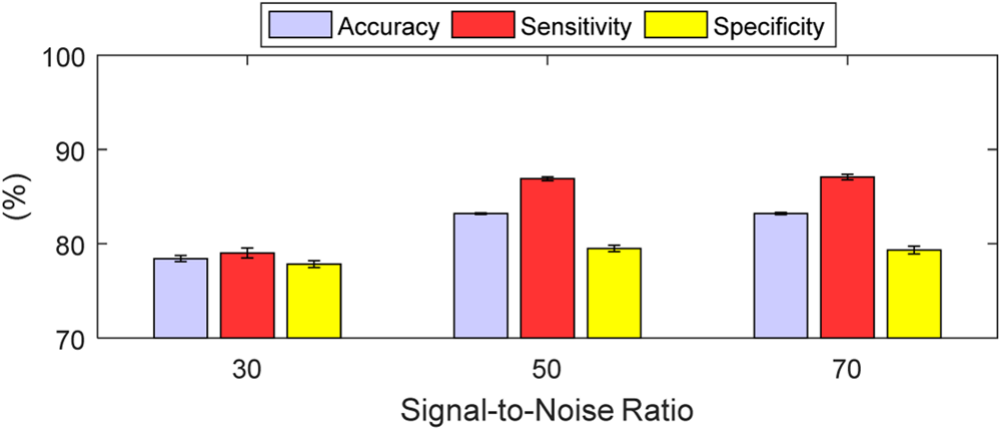
The performance metrics of Linear SVM for the realistic test scenarios (TC 4) withheld from the classifier. The realistic test scenario includes variations in bladder volume, urine conductivity, and FEM boundary size. The performance metrics are in terms of the mean and standard deviation of accuracy, sensitivity, and specificity. Little difference in accuracy is seen between 50 dB and 70 dB. However, the accuracy of Linear SVM decreases by 4.77% from 50 dB to 30 dB.

To investigate the reason for the drop in the performance, we analysed the Linear SVM, which performed consistently well throughout each TC. A higher percentage of bladder volumes were misclassified around the threshold 300 ml, than in the previous three TCs (15.08% vs. < 4%). The reason for the higher misclassification around the threshold is that we added measurements with volume within ±5 ml of the threshold to determine how bladder volumes near the threshold with noise would be classified. At bladder volumes of 295 ml and 305 ml, 72% and 25.15% test set observations were misclassified, respectively, compared to 72.58% and 27.43% at 299 ml and 301 ml, respectively. Excluding the volumes in the immediate vicinity of the threshold reduces the percentage misclassified to rates to a level similar to those obtained in TC 3. More observations were misclassified at 30 dB for the Linear SVM than at 50 dB or 70 dB, as can be seen in Fig. 9. The contribution of variations in urine conductivity and in the FEM boundary to misclassification rates was similar to that of the previous TCs. From the analysis, the SNR and the misclassification of the bladder state around the threshold were the most significant factors.

The SNR effect can be minimized by suitable design of an EI measurement system. One way to compensate for the misclassification around the bladder state threshold is to use multiple thresholds: thresholds where there is high confidence of the bladder being full or not full and a region between where there is uncertainty as to the state. Dependent on the application, the point of alert can then be selected more reliably and more accurately. For less than or equal to 260 mL, at least 98% of the time the classification is correct. Thus, if we set a threshold at the 240 ml bladder volume we could be very confident in classifiers correctly identifying a not full bladder. Depending on the clinical application, if it is important to correctly identify the full bladder, a threshold could be set at 360 ml, and the result would be correct at least 99% of the time. Probabilistic methods such as relevance vector machines could enhance the prediction accuracy within the uncertainty region.

### Phantom Results and Discussion

**Figure 10:**
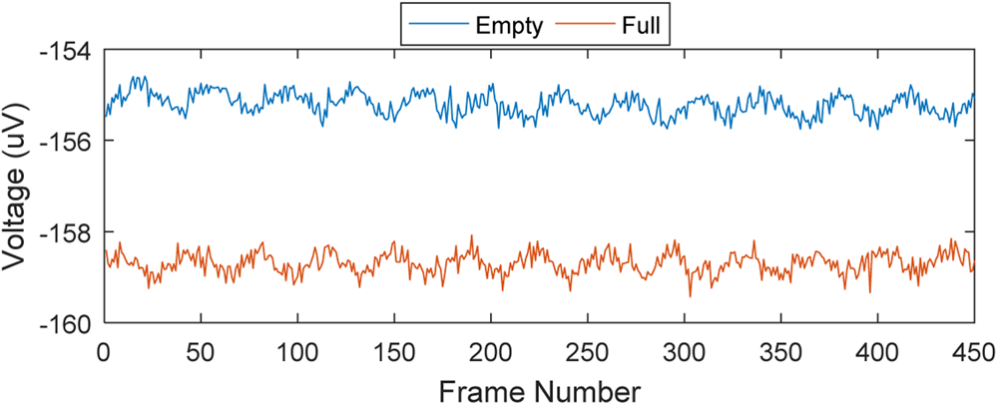
Analyzing empty vs. full experimental voltage measurements across all frames for the 10^th^ injection pattern and 1^st^ measurement index. The full and empty voltage curves can easily be separated, aiding classification.

Classification was also performed on the realistic pelvic phantom, with a range of appropriate bladder volumes. For each of the phantom bladder volumes, all classifiers predicted the bladder state with 100% accuracy. Analysing many of the measurement locations for a specific pattern, the voltage values for empty and full are separable, an example of which is shown in Fig. 10. Modern day EI measurement devices such as the Swisstom Pioneer Set have SNRs over 60 dB^30^. An EI capturing system with a high SNR would explain the high classification accuracy with phantom data.

The significant experimental result of 100% accuracy confirms the simulated outcomes for high SNR levels, and demonstrates the strong promise of classification for bladder state monitoring in a real-life scenario. Future work will include more volumes around the threshold.

### Study Caveats

This study makes a number of assumptions that include: high volumes of bladder volume data exist for patients and the data for multiple patients are applicable to other patients. However, as the complexity of the TCs increases, the number of observations for each simulation candidate is decreased, making the experiment more realistic. In terms of gathering a suitable amount of data in real life, large numbers of datasets could indeed be gathered from an individual or small number of patients quickly. Adaptive techniques to adjust the threshold may also cater for patient-to-patient adaption of the classification algorithms.

We also assumed the bladder shapes were similar for each patient regardless of gender, as very little information is available in the literature relating the dimensions of the bladder, the filling volume and gender differences. By setting a uniform background conductivity of 0.2 S m^−1^, the background conductivities were controlled allowing us to examine each factor in detail. However, it is reasonable to assume that if classification is successful with the range of bladder volumes, positions, and conductivities tested here, that the technique has good potential to work regardless of gender-based variations in bladder size and position.

Dependent on the application, it may be important to prioritize either the sensitivity or the specificity. For example, in UI where it is important to alert the user before voiding, a high sensitivity is desired. This could bias the choice of the classifiers, where the SVM classifiers generally had a higher sensitivity than the KNN classifiers. In applications where it is important to alert the patient of a full bladder before voiding, the sensitivity should be maximized. Future work will also focus on fine-tuning classifiers based on the clinical need.

Opportunities to further improve the results presented here exists, such as tuning the number of neighbours in Cosine KNN and SVM hyperparameters using *n*-fold cross-validation on the training data. Further work will explore the listed areas as well as increasing the complexity of phantom experiments and catering for other electrical impedance measurement challenges such as electrode contact and movement.

Future work includes human clinical testing to assess the effect of the presented factors as well as uncontrollable or unknown factors such as electrode contact on classification.

## Conclusion

This paper has presented a novel method for determining bladder state to aid patients with UI in their everyday lives. Specifically, in this work, we have demonstrated for the first time the successful classification of ‘full’ and ‘not full’ bladder state using electrical impedance measurements and simulation data. Specifically, we first investigated the effects of changing the simulated SNR, bladder volume, urine conductivity, and patient boundary on the electrical signal classification of bladder state in simulation. We compared the performance metrics of the chosen machine learning classifiers, support vector machines and k-Nearest-Neighbour. We found the SNR and bladder volumes around the bladder state threshold had the most significance effects on the performance of the classifiers. Linear SVM was the most transferable to withheld scenarios when tested on a more realistic simulation case. We demonstrated that a Linear SVM classifier performed with 81.66 ± 0.12% accuracy on previously unseen simulated test scenarios spanning variations in bladder volume, urine conductivity, and pelvic boundary size. Furthermore, classification on experimental data recorded using a pelvic phantom and bladders with realistic shape and conductivity profiles achieved 100% accuracy in differentiating between the investigated full and not full bladder states. These results highlight the feasibility of using classification in the real world under more complex scenarios. This result promises to support the development of tools to aid patients with urinary incontinence in their everyday lives.
